# Characterization of an Ultrasonic Local Positioning System for 3D Measurements

**DOI:** 10.3390/s20102794

**Published:** 2020-05-14

**Authors:** Khaoula Mannay, Jesús Ureña, Álvaro Hernández, Mohsen Machhout, Taoufik Aguili

**Affiliations:** 1 EμE Lab, Faculty of Sciences of Monastir, National Engineer School of Tunis, University of Tunis EI Manar, B.P. 37, Le Belvédère, Tunis 1002, Tunisia; khaoula.mannay@edu.uah.es; 2 Electronics Department, University of Alcala, Alcalá de Henares, E-28805 Madrid, Spain; alvaro.hernandez@uah.es; 3 EμE Lab, Faculty of Sciences of Monastir, University of Monastir, Monastir 5019, Tunisia; mohsen.machhout@fsm.rnu.tn; 4 SysCom Lab, National Engineer School of Tunis, University of Tunis EI Manar, B.P. 37, Le Belvédère, Tunis 1002, Tunisia; taoufik.aguili@gmail.com

**Keywords:** 3D positioning, ultrasonic sensory systems, local positioning systems (LPS), LMS, multilateration, maximum likelihood estimation (MLE)

## Abstract

Indoor location and positioning systems (ILPS) are used to locate and track people, as well as mobile and/or connected targets, such as robots or smartphones, not only inside buildings with a lack of global navigation satellite systems (GNSS) signals but also in constrained outdoor situations with reduced coverage. Indoor positioning applications and their interest are growing in certain environments, such as commercial centers, airports, hospitals or factories. Several sensory technologies have already been applied to indoor positioning systems, where ultrasounds are a common solution due to its low cost and simplicity. This work proposes a 3D ultrasonic local positioning system (ULPS), based on a set of three asynchronous ultrasonic beacon units, capable of transmitting coded signals independently, and on a 3D mobile receiver prototype. The proposal is based on the aforementioned beacon unit, which consists of five ultrasonic transmitters oriented towards the same coverage area and has already been proven in 2D positioning by applying hyperbolic trilateration. Since there are three beacon units available, the final position is obtained by merging the partial results from each unit, implementing a minimum likelihood estimation (MLE) fusion algorithm. The approach has been characterized, and experimentally verified, trying to maximize the coverage zone, at least for typical sizes in most common public rooms and halls. The proposal has achieved a positioning accuracy below decimeters for 90% of the cases in the zone where the three ultrasonic beacon units are available, whereas these accuracies can degrade above decimeters according to whether the coverage from one or more beacon units is missing. The experimental workspace covers a large volume, where tests have been carried out at points placed in two different horizontal planes.

## 1. Introduction

In recent decades, indoor positioning systems have spread worldwide due to the huge number of contexts and smart applications where they can be applied, such as personalized healthcare, safety care, intelligent monitoring, tracking, context-aware and location-based services, etc. These systems are often based on different sensory technologies, such as radiofrequency, infrared, ultrasounds, magnetic fields, visible light, etc., acquired by a specific receiver [1,2,3]. The technology choice depends on some parameters: the requested accuracy, the cost of the system, the final application, or the positioning algorithm involved, among others [4,5]. In this way, recently, some commercial systems based on Ultra-Wide Band (UWB) signals have successfully achieved positioning errors in the range of decimeters [6,7], with an operation mode based on the determination of times-of-arrival and measuring distances from the LoS path. They often operate at longer distances, with an omnidirectional emission, but they also involve certain limitations [8].

Ultrasonic systems have already been considered as an interesting technology for indoor applications, mainly due to some advantages, such as low power, suitable accuracy under line-of-sight (LoS) conditions, or even low cost, particularly when considering the hardware devices and equipment involved in practical real-time implementations [9,10,11].

Some 3D ultrasonic positioning systems have been developed previously using different configurations, as summarized in [12,13]. They are based on two main approaches: emitters are placed at fixed positions whereas receivers move in the environment, and vice versa. To estimate the receivers’ positions, a trilateration method is used in most cases [5,14,15], often based on the determination of time-differences-of-arrival (TDOA) [5], times-of-arrival (TOA) [12], angle-of-arrival (AOA) [14], or even on hybrid techniques [16], to measure the distances between emitters and receivers. It is worth noting that, if several position estimates are obtained, then the final resulting position is often defined as the mean or the center of all the previous estimates [13,16].

With regard to the first approach, where the emitters are placed at known positions and the receivers are mobile, different 3D ultrasonic positioning systems have been developed with diverse configurations, numbers of beacons and positioning techniques. Some emitter configurations are composed by a set of beacons installed in the ceiling as in [15]. It presents a set of six synchronized beacons in the ceiling, pointing to the center of the room to measure TDOAs. Other configurations consist of beacons installed at the ceiling corners [17], where three independent beacons, synchronized with the receiver, are used for a hybrid technique based on AOAs and TOAs; similarly, the deployment of four beacons at the ceiling corners is proposed in [18]. Furthermore, four beacons are synchronized with a fixed microphone in [12], in order to measure the distances between the emitters and the receiver. Furthermore, in [5,19] a set of beacons are slightly shifted from the same background plane, while oriented towards the common coverage zone. Nevertheless, these configurations with all the beacons almost in the same plane may often imply some limitations when measuring distances in the perpendicular direction to that plane. To overcome this constraint, some previous works have also proposed the deployment in parallel or different planes [16,20].

As for the second approach, in [21] a 3D positioning system is proposed, based on five ultrasonic emitters attached to a mobile user, whereas a set of five ultrasonic receivers is fixed at known points. This system uses a trilateration technique and the extended phase accordance method as a tracking algorithm to estimate the distance to the mobile object, as well as a time division multiple access (TDMA) method as a communication link, so a trigger pulse synchronizes the emitters and the receivers. This proposal is evolved in [22], by defining a receiver with four coplanar beacons placed perpendicularly to the mobile emitters. Similarly, another 3D system composed by a single mobile emitter and a set of six fixed and coplanar receivers at known positions is designed in [23]. It uses a linear ultrasonic chirp and the phase correlation approach to determine the corresponding TOAs, together with a spherical trilateration technique to calculate the position estimates.

In this work, a 3D ultrasonic local positioning system (ULPS) for indoor environments is proposed, implemented and validated, confirming that the proposal is a suitable 3D solution with high accuracy in volumes with a proper coverage. This definition of this ULPS becomes particularly suitable for 3D environments, and it is partially based on a previous system called LOCATE-US [11,24]. It proposes a deployment scheme of the beacons to minimize positioning errors in the common coverage area, whereas the encoding applied to transmissions allow the simultaneous determination of difference-times-of-arrival without synchronization between beacons and receivers. Furthermore, since multiple beacons will provide several position estimates, this work also proposes a suitable integration of the estimates into a final position, based on a minimum likelihood estimation (MLE) approach. Another relevant novelty is that all the signal processing proposed, as well as the deployment of the ULPSs, have been experimentally validated, compared to a preliminary study, based on simulation for 3D positioning [25]. This validation includes a complete set of results with 3D-positioning measurements, considering 3D-arranged receivers to improve the reception of signals coming from very different angles of arrival. 

The rest of the manuscript is organized as follows. Section 2 provides a general overview of the 3D positioning system. It is based on the deployment of three LOCATE-US ULPS prototypes in perpendicular planes of the environment, whereas a receiving module with three microphones allows us to capture transmissions coming from opposite directions. Both prototypes, beacons and receivers, with their main features, are described in detail in Section 2, as well as the proposed ultrasonic signal processing based on encoding techniques. Section 3 shows the proposal’s performance in simulation when only one ULPS module is used to cover the area under study. Since only the beacons from one ULPS are available, the results present some limitations on coverage and accuracy, particularly for the perpendicular direction to the plane where the beacons are installed. In order to tackle these drawbacks, Section 4 extends the study to the proposal based on three ULPSs, thus providing simulations that verify that the proposal achieves similar accuracies in any direction and increases the coverage area with lower errors. Section 5 presents some experimental results, which, under similar circumstances and at the same points under analysis, evaluate the real performance of the proposal, obtaining consistent figures with those from simulations. Finally, conclusions are discussed in Section 6.

## 2. Ultrasonic Local Positioning System Overview

### 2.1. Technical Description and 3D Configuration

**Figure 1:**
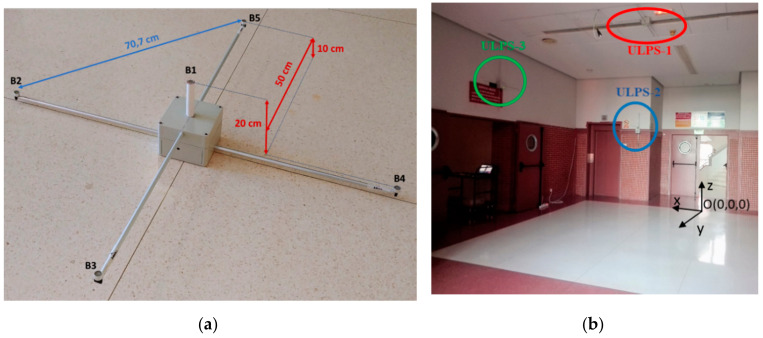
(**a**) General view of the LOCATE-US 3D ultrasonic local positioning system (ULPS) developed by the GEINTRA-US/RF research group from the University of Alcala [11]; (**b**) Configuration proposed for the 3D positioning system, based on three ULPSs installed on three perpendicular planes in the experimental environment, where the point O is the origin of coordinates.

This proposal is based on the beacon unit developed for the LOCATE-US prototype, designed and implemented by the GEINTRA-US/RF Research Group from the University of Alcala [11,24]. It is a compact, and lightweight ultrasonic beacon architecture. It is formed by five ultrasonic transducers located at the four corners and at the center of a square with a side of 0.707 m, as can be observed in Figure 1a. The ultrasonic transducers are controlled by a field-programmable gate array (FPGA)-based platform that consumes an average power of 3.3 W. Note that the beacons are installed at fixed positions and normally can be plugged to the mains, so the consumption is not critical. The five ultrasonic transducers have the same orientation and cover a similar volume size. This coverage volume is actually a truncated cone of 53 m^3^ when the height between the small base (the circle including the ULPS with an area about 0.78 m^2^) and the large base (a circle about 40 m^2^ on the ground) is 3.5 m, assuming that the effective emission angle of transducers is around 120°. The ultrasonic transducer used is the PROWAVE 328ST160 [26], which has a total beam angle of 100° (−6 dB) measured at 32.8 kHz and can be extended to 120° with a small loss (around −7 dB). As its internal diameter is 13.2 mm, it is expected that the beam angle decays at higher frequencies (at 40 kHz roughly the wavelength drops from 10.4 mm to 8.5 mm). The five beacons *Bj*, *j* = 1,2, …, 5, are not coplanar, since they have a small variation in height to improve the coordinate estimation in this direction. Beacons B3 and B5 are placed at 10 cm high, B1 is 20 cm high, and B2 and B4 remains in the background plane (normally the ceiling or a wall) [8]. Inside this common coverage space, different receivers can estimate their positions independently and autonomously [27]. According to the transducer datasheet [26], this ultrasonic transducer has a resonance peak at 32.8 kHz with a bandwidth of 2.5 kHz; nevertheless, in this work, we are interested in the middle zone between this resonance frequency and the other one existing at 46 kHz (the transducer has been tested at these higher frequencies to determine its behavior, see [28] for further details). Using a central frequency of 41.667 kHz, a bandwidth of 8 kHz can be effectively used. Furthermore, the ultrasonic transmissions are encoded with different 255-bit Kasami sequences [29], due to their suitable cross-correlation and auto-correlation properties. These sequences are Binary Phase Shift Keying (BPSK) modulated to fit the available bandwidth. The ultrasonic transmission period is 50 ms, in order to discard possible multipath effects between successive transmissions. As these are encoded, the medium access technique can be either code division multiple access (CDMA) or time division multiple access (TDMA). Hereinafter, the technique applied is T-CDMA (that is, CDMA with a certain time separation between the emissions from different beacons to avoid a complete overlapping of emissions). 

Nevertheless, it is worth noting that this LOCATE-US ULPS presents limitations for 3D positioning, mainly in the direction of the ultrasonic transmissions (perpendicular to the plane at which the beacon unit is installed), where the dispersion of the obtained position is high, providing significant errors for further distances and under adverse conditions [24,27,30].

To overcome this drawback in 3D indoor positioning, this work proposes the deployment of several ULPSs in orthogonal planes, so the final performance depends on the number of ULPSs and their emission patterns. This proposed setup provides an enhancement of accuracy in the position estimation for any direction/coordinate. Particularly, it installs three beacon units (ULPS-1, ULPS-2, and ULPS-3) in three perpendicular planes: ULPS-1 on the ceiling, and ULPS-2 and ULPS-3 on two perpendicular walls, thus following typical shapes in indoor spaces, as is shown in Figure 1b. The scanned environment can be divided into four zones: one covered by the three ULPSs (in the center of the room), zones with the different combinations of two ULPSs (ULPS-1 + ULPS-2, ULPS-1 + ULPS-3, ULPS-2 + ULPS-3), zones covered just by one ULPS (ULPS-1, ULPS-2 or ULPS-3); and zones that are not covered by any ULPS within the perimeter of the room. Note that the positioning accuracy depends on those ULPS’s coverage combinations. This work has been focused on the experimental environment shown in Figure 1b: it is a university hall, thus a large space of 7 × 8 × 3.5 m^3^. Table 1 shows the coordinates of the central beacons B1 in Figure 1a, for every ULPS; note that ULPS-2 and ULPS-3 have different heights and are not placed at the centers of the wall. It is worth noting that, in general terms, acoustic local positioning systems (LPSs) present a performance that may strongly depend on the environment complexity, where multi-path effect or non line-of-sight (NLoS) situations can rapidly degrade the correct estimation of TOAs or TDOAs, thus implying higher positioning errors. Under these circumstances, the proposal described hereinafter results in a more robust solution due to the fact that 15 ultrasonic emitters are placed in three different and orthogonal planes. This makes it feasible to have available a large enough number of LoS measurements to operate even in complex environments. Additionally, as will be detailed in Section 2.2, the low-level processing algorithms applied to the received signals deal well with the multipath effect and low signal-to-noise ratios.

In this situation, fifteen ultrasonic transducers are transmitting simultaneously, and any receiver can process these emissions to determine the corresponding TDOAs, and then estimate its own position by applying a hyperbolic positioning algorithm. The five beacons of each ULPS are controlled by an FPGA-based circuit, which operates at 5 V. It performs three main tasks: standby/monitoring, Ethernet configuration and ultrasonic transmissions. The current consumption is 0.67 A, 0.66 A and 0.68 A for each task, respectively. Note that the beacons are installed at fixed positions and normally can be plugged to the mains, so the consumption is not critical. 

**Figure 2:**
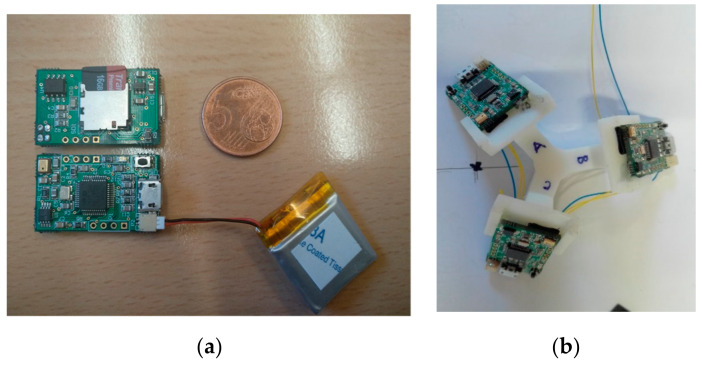
General aspect of the reception module: *(***a**) a single ultrasonic receiver; (**b**) a multiple receiver prototype.

With regard to the reception stage, a multiple ultrasonic prototype has been considered in this work, involving three independent ultrasonic receivers. As can be observed in Figure 2a, a single ultrasonic receiver consists of an omnidirectional microphone MEMS PU0414HR5H-SB (which includes the emitters’ bandwidth around 41.67 kHz, with 94 dB SPL at 1 kHz) [31], a high-pass filter to discard audible frequencies, an analog-to-digital converter (ADC) sampling the received signals at 100 kHz, and a STM32F103 processor unit. This single ultrasonic receiver is capable of acquiring a data window with a length of up to 100 ms, thus implying a global update rate of 10 Hz. Due to the geometry of the beacon units, this single receiver provides good performance when it is oriented to a certain ULPS and installed in its parallel plane, so it is possible to acquire the five ultrasonic transmissions involved. The problem arises when there are three beacon units available in the environment with completely different orientations, so that a single receiver will not be able to receive all the fifteen transmitted signals in many cases. The single receiver or the 2D receiver is used for 2D positioning when the emitters are placed on the ceiling and the receiver is installed, horizontally, e.g., on board of a mobile robot on the floor (the aperture beam of the receiver is about 180°). However, in the 3D configuration, three ULPSs are placed in three perpendicular walls. The use of this receiver does not guarantee the reception of all the transmissions, especially the reception from the ULPSs emitting from behind. This is the reason why a multiple receiver prototype has been proposed. It is composed of three receivers (namely RA, RB and RC) placed on the upper faces of a tetrahedron, as can be observed in Figure 2b (the inclination angle is 60° for each receiver). Each microphone captures the signals coming from the ULPSs in front of it. This structure aids in maximizing the probability of acquiring up to fifteen signals coming from the three beacon units, thus improving the performance in the position estimation [32,33]. The 3D receiver consumes 80 mW roughly (24 mA at 3.3 V), if it is continuously acquiring signals.

For this 3D configuration, the five beacons of each ULPS emit simultaneously (or with known delays between them) and, then, the receiver does not need to be synchronized with the beacons if we use hyperbolic positioning (multilateration). Furthermore, if each microphone processes its TDOAs in an independent way (as it is the case here), there is no need for synchronization between the microphones. The reason to provide a synchronization among microphones is for those cases where it is desired to apply a unique positioning algorithm with all the TOA measurements (no matter which microphone received the signals). Note that the same processing module is used for the three microphones with a master clock of 50 MHz and an acquisition frequency of 100 kHz (the minimum resolution step in the TDOA determination is then 10 µs). For this reason, any consideration about the time jitter in the duration of each processing window is negligible.

### 2.2. Proposed Signal Processing

**Figure 3:**
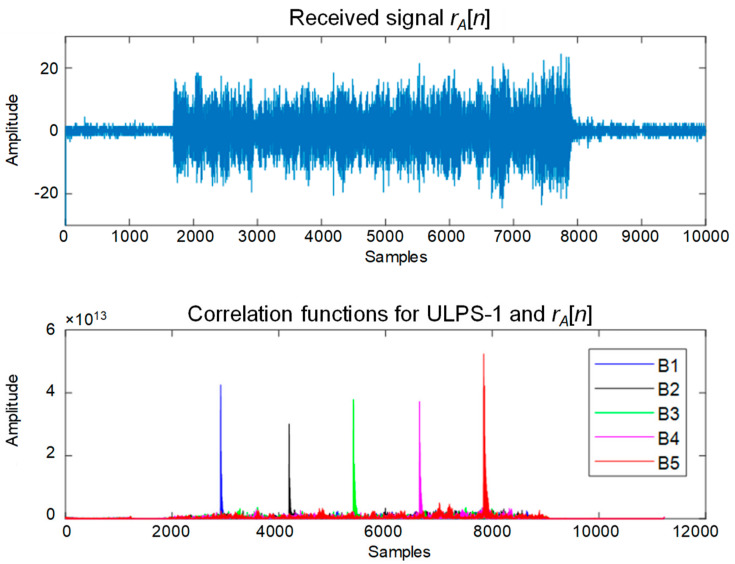
Example of the received signal *r_A_*[*n*] (**top**) and the five correlation functions for ULPS-1 (**bottom**).

In the case mentioned before, with three different ULPSs deployed in the environment and the multiple receiver prototype, three incoming received signals, *r_A_*[*n*], *r_B_*[*n*] and *r_C_*[*n*] are acquired, each one containing up to fifteen ultrasonic transmissions. Afterwards, for each one of these three acquired signals, fifteen correlations (45 correlations globally) are performed, involving the different fifteen 255-bit Kasami sequences used to encode the ultrasonic emissions. These correlation functions and their maximum peaks are used to determine a set of TDOAs. Figure 3 presents an example of the five correlation functions that are obtained at the receiver RA only for ULPS-1, using different colors for each beacon B_j_. As was mentioned before, the window size of the received signal *r_A_*[*n*] is 10.000 samples, corresponding to 0.1 s long. This size allows at least one emission period to be captured.

From the peaks obtained, the TDOAs can be easily obtained, by taking one of the beacons (e.g., beacon B1) as reference. Finally, by multiplying these TDOAs by the speed of sound in air, we obtain the distance differences used in the hyperbolic positioning algorithm. 

The position of each receiver is estimated from these distance differences using a Gauss–Newton algorithm, which implies:

- Defining an initial position $p_{0}$ for the receiver (it should be chosen according to the a priori knowledge of the environment—In our case we consider the center of the positioning area). In the following steps of the algorithm, this position will be the previously obtained $p_{k-1}$.
- Minimizing the following function *F*(*x*, *y*, *z*):
  (1) $$F\left(x,y,z\right)=\overset{n}{\underset{j=2}{\sum }}{\left(\hat{∆}r_{1j}-∆r_{1j}\right)}^{2}=\overset{n}{\underset{j=2}{\sum }}{\left[f_{j}\left(x,y,z\right)\right]}^{2}$$
  where $\hat{∆}r_{1j}={\hat{r}}_{1}-{\hat{r}}_{j}$ are the theoretical distance differences computed at the last position of the receiver between the reference beacon B1 and the others B*j* (*j* = 2, …, n); and $∆r_{1j}$ are the same distance differences, but measured. Note that:
  (2) $$\frac{\partial F}{dx}=2\cdot \sum_{j=2}^{n}f_{j}\cdot \frac{\partial f_{j}}{dx};\;\frac{\partial F}{dy}=2\cdot \sum_{j=2}^{n}f_{j}\cdot \frac{\partial f_{j}}{dy};\;\frac{\partial F}{dz}=2\cdot \sum_{j=2}^{n}f_{j}\cdot \frac{\partial f_{j}}{dz}$$

Finally, the variations along each axis can be computed:

(3) $$X={\left(A^{T}A\right)}^{-1}A^{T}B$$

where with:

(4) $$X=\left(\begin{array}{c} \frac{\partial F}{\partial x}\\ \frac{\partial F}{\partial y}\\ \frac{\partial F}{\partial z} \end{array}\right);\;\;B=\left(\begin{array}{c} \begin{array}{c} f_{1}\\ f_{2}\\ \ldots \end{array}\\ f_{n} \end{array}\right)\;\;\;A=2J=2\left(\begin{array}{c} \begin{array}{ccc} \frac{\partial f_{1}}{\partial x} & \frac{\partial f_{1}}{\partial y} & \frac{\partial f_{1}}{\partial z}\\ \frac{\partial f_{2}}{\partial x} & \frac{\partial f_{2}}{\partial y} & \frac{\partial f_{2}}{\partial z}\\ \ldots & \ldots & \ldots \end{array}\\ \begin{array}{ccc} \frac{\partial f_{n}}{\partial x} & \frac{\partial f_{n}}{\partial y} & \frac{\partial f_{n}}{\partial z} \end{array} \end{array}\right)$$

- Estimating, at each step *k*, the new position $p_{k}=p_{k-1}+∆X$, and repeating the process until ∆X becomes small enough (according to a pre-defined threshold).

Consequently, for any particular test point P, applying this algorithm at each receiver for each one of the three ULPSs, it is possible to obtain up to three different estimated positions for each receiver, (*x*_1_*, y*_1_*, z*_1_), (*x*_2_*, y*_2_*, z*_2_) and (*x*_3_*, y*_3_*, z*_3_). Note that every ULPS has beacons emitting different codes and, consequently, the receiver is able to discriminate and calculate a position for each ULPS (if a large enough number of distance differences is obtained). Furthermore, the model of noise propagation for each coordinate (σ*_x_*, σ*_y_* and σ*_z_*) in a single ULPS can be previously determined, depending on the position obtained, by simulation and empirical tests (see Section 3). These typical deviations are obtained from values previously stored for a grid of discrete positions for every ULPS, and used later for the position fusion. Then, for three ULPSs, nine positioning errors can be modelled, three per coordinate, so, for example, in the coordinate *x*, the positioning errors can be: p(*x*_1_*|x*) = N(*x*,σ_1*x*_^2^), p(*x*_2_*|x*) = N(*x*,σ_2*x*_^2^) and p(*x*_3_*|x*) = N(*x*,σ_3*x*_^2^), where N(*x*,σ*_x_*^2^) is the normal distribution with mean *x* and variance σ*_x_*^2^. The final estimated coordinate *x_MLE_* is shown in Equation (5), as well as for *y_MLE_* and *z_MLE_*.

(5) $$x_{MLE_{}}=\frac{\sigma_{1x}^{-2}\cdot x_{1}+\sigma_{2x}^{-2}\cdot x_{2}+\sigma_{3x}^{-2}\cdot x_{3}}{\sigma_{1x}^{-2}+\sigma_{2x}^{-2}+\sigma_{3x}^{-2}}\;y_{MLE_{}}=\frac{\sigma_{1y}^{-2}\cdot y_{1}+\sigma_{2y}^{-2}\cdot y_{2}+\sigma_{3y}^{-2}\cdot y_{3}}{\sigma_{1y}^{-2}+\sigma_{2y}^{-2}+\sigma_{3y}^{-2}}\;z_{MLE_{}}=\frac{\sigma_{1z}^{-2}\cdot z_{1}+\sigma_{2z}^{-2}\cdot z_{2}+\sigma_{3z}^{-2}\cdot z_{3}}{\sigma_{1z}^{-2}+\sigma_{2z}^{-2}+\sigma_{3z}^{-2}}$$

A new standard deviation σ*_x_* can be defined for coordinate *x* after the fusion (and similarly for σ*_y_* and σ*_z_*), according to Equation (6).

(6) $$\sigma_{x}^{-2}=\sigma_{1x}^{-2}+\sigma_{2x}^{-2}+\sigma_{3x}^{-2}$$

**Figure 4:**
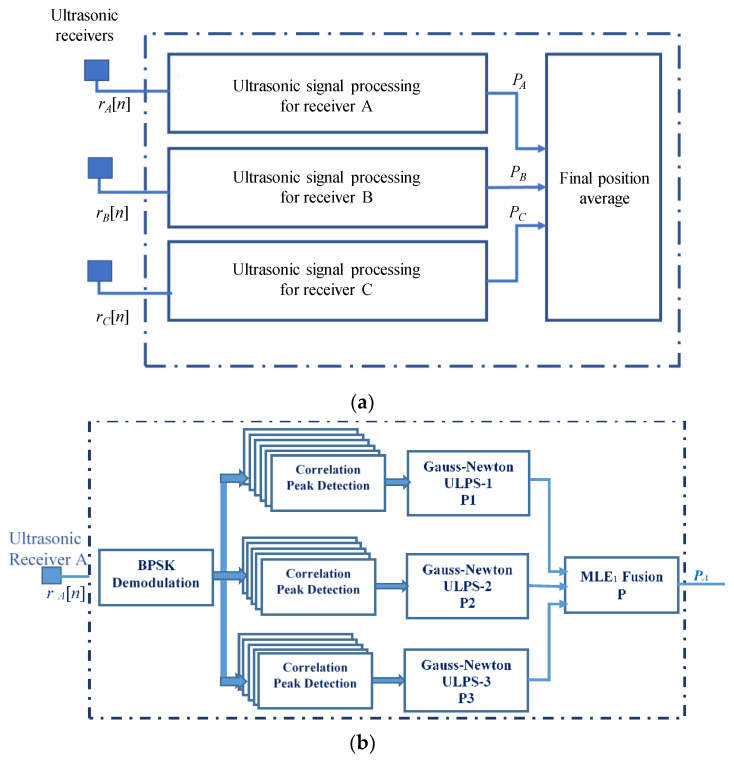
(**a**) General block diagram of the processing proposed for the multiple receiver prototype; (**b**) detailed block diagram for receiver RA.

Note that the coordinates (*x_MLE_*, *y_MLE_*, *z_MLE_*) can be obtained for each receiver RA, RB and RC, resulting in three positions *P_A_*, *P_B_* and *P_C_*. These three resulting positions are averaged to obtain the final estimated position. This whole processing is detailed in Figure 4a, with three identical branches, each one corresponding to an ultrasonic receiver. On the other hand, Figure 4b particularizes the details of the processing algorithm for the first receiver RA. This processing is also the same for receivers RB and RC. It is worth noting that the availability of nine position estimates at the input of the MLE fusion module is optimistic. In real cases, due to geometrical considerations, coverage areas, noise, and other constraints, some ultrasonic transmissions will not be detected at the receivers, thus posing a challenge to obtain the aforementioned nine estimates.

It is worth highlighting that the positions to fuse are estimated for each ULPS and not including all the distances from all the ULPSs simultaneously (making a fusion at distance level), because each ULPS operates without a precise synchronization with the others.

Generally speaking, the positioning of any receiver should be carried out in real time. Nevertheless, in order to use the MLE fusion algorithm, the variances (σ*_ix_*^2^, σ*_iy_*^2^, σ*_iz_*^2^) of the estimated positions in the X, Y and Z axis, where *i* = {1, 2, 3} is the index of the ULPS *i*, must be known for a set of different ground-truth positions. For that, the final application of the proposal requires a training phase, where the aforementioned variances are obtained off-line for the volume under analysis. These variances are used later during real-time operation to estimate the receiver’s position in the MLE fusion.

A last aspect to be considered is the computational load determined by the proposed signal processing. It requires the calculation of three demodulation processes for three input buffers, *r_A_*[*n*], *r_B_*[*n*] and *r_C_*[*n*], with a length of 10,000 samples, and a two-samples demodulation symbol. Afterwards, a total of 45 correlations are implemented, actually 15 per each demodulated signal, where the demodulated signal is still 10,000 samples long and the pattern sequence, assigned to each transmitter, has 255 samples, corresponding to the 255-bit Kasami codes applied to the transmission encoding. A peak detector is performed in the resulting correlated signal, whose peaks are used to determine the partial positions *P_A_*, *P_B_* and *P_C_*. It is worth mentioning that some previous works have already dealt with this challenge [34], often using SoC (System-on-Chip) architectures based on FPGA devices to take advantage of the parallelism from the configurable logic and the flexibility from the available processors. For this work, the STM32F103 processor unit manages the acquisition system and the communication with a high-level device (Personal Computer PC, smartphone or tablet) via an USB port. So, all the proposals and tests have been validated hereinafter without real-time constraints.

## 3. Positioning Performance for a Single ULPS

One of the main quality parameters to evaluate an indoor positioning system is accuracy. This depends on some factors, such as the system’s geometry, the distribution and number of beacons, or some adverse effects (multipath, etc.). The positioning error caused by the geometric distribution of the ULPS units can be estimated by the position dilution of precision (PDOP) for a 3D approach. The PDOP can be decomposed as combination of the horizontal dilution of precision (HDOP) and the vertical dilution of precision (VDOP). In addition to a mathematical derivation, according to [35], it can be also estimated heuristically by using the covariances, *σ_x_*^2^, *σ_y_*^2^ and *σ_z_*^2^, for the three coordinates (*x*, *y*, *z*) of a position *P*, as shown in Equation (7).

(7) $$PDOP\approx \frac{\sqrt{\sigma_{x}^{2}+\sigma_{y}^{2}+\sigma_{z}^{2}}}{\sigma_{m}}$$

where *σ_x_*^2^, *σ_y_*^2^, *σ_z_*^2^ are the coordinate variances of the estimated position for X, Y and Z axes; and σ_m_ is the standard deviation of the ultrasonic distance measurements (it is experimentally fixed at σ_m_ = 0.01 m). Note that the smaller the PDOP value is, the better the position accuracy is. 

**Figure 5:**
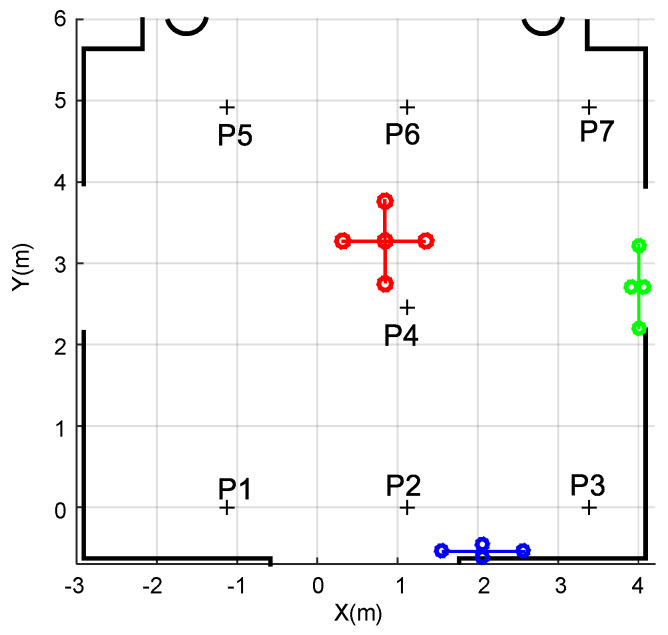
Environment and grid of positions to be considered hereinafter in the evaluation of the positioning performance by simulation.

Particularly for the LOCATE-US ULPS, already described in Section 2, its accuracy decreases as the receiver gets further away from the center of the coverage area in the perpendicular direction to the ULPS. Simulation tests have been carried out only for ULPS-1 (the red one on the ceiling in Figure 5), according to the coordinates aforementioned in Table 1. For that purpose, a grid of receiver’s positions P1–P7 has been considered at two different heights (*z*_1_ = 1.35 m and *z*_2_ = 1.93 m), as can be observed in Figure 5. Note that, in the analysis developed at this stage, the points are selected to cover a large area, where each point represents a particular situation regarding the global coverage of the assembly.

**Figure 6:**
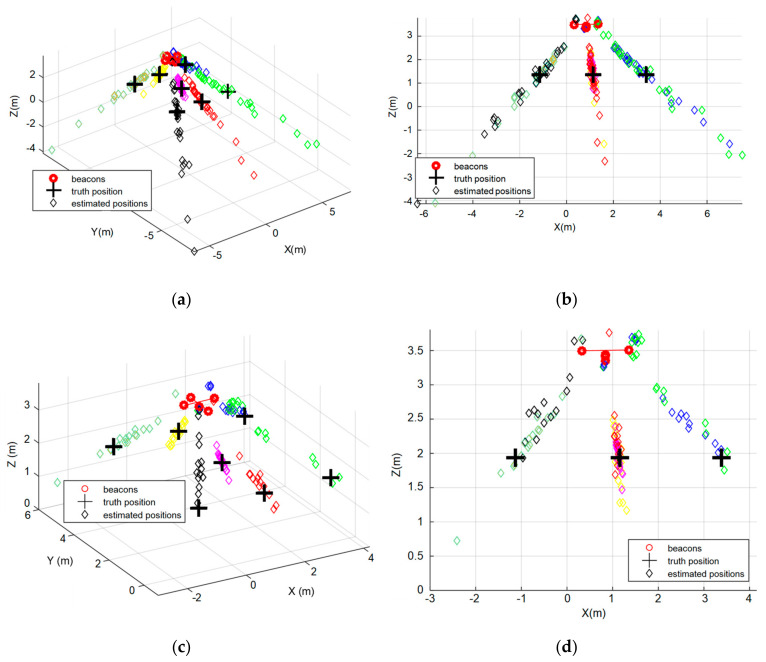
Positions estimated by simulation for ULPS-1 with the 3D receiver prototype (a different color for each simulated position P1–P7): (**a**) 3D representation for *z*_1_ = 1.35 m; (**b**) XZ projection for *z*_1_ = 1.35 m; (**c**) 3D representation for *z*_2_ = 1.93 m; (**d**) XZ projection for *z*_2_ = 1.93 m.

At each position P1–P7, thirty simulations have been carried out, using hyperbolic trilateration with the Gauss–Newton positioning algorithm described before. Figure 6 shows the simulated results for ULPS-1 at both heights *z*_1_ and *z*_2_. Note that the different positions P1–P7 have been distinguished by using different colors. For example, the black diamonds correspond to the estimated positions for P1, which spread around the ground truth, with a significant dispersion along the ultrasonic emission direction. This uncertainty in the determination of coordinate *z* (the perpendicular axis to the plane where the ULPS-1 is installed) increases when the distance between the ULPS and the receiver does. This is why those results for *z*_2_ in Figure 6c,d present more concentrated clouds of points, especially with regard to the coordinate *z*. For the same reason, ULPS-1 is suitable for the estimation of coordinates x and y, with a better performance for the points in the center of the coverage zone, such as point P4 (pink diamonds in Figure 6). The same behavior can be derived for ULPS-2 and ULPS-3 that are installed on two perpendicular walls, so their highest dispersions are in the *y*-axis and the *x*-axis, respectively. Table 2 and Table 3 resume the main values from Figure 6.

**Figure 7:**
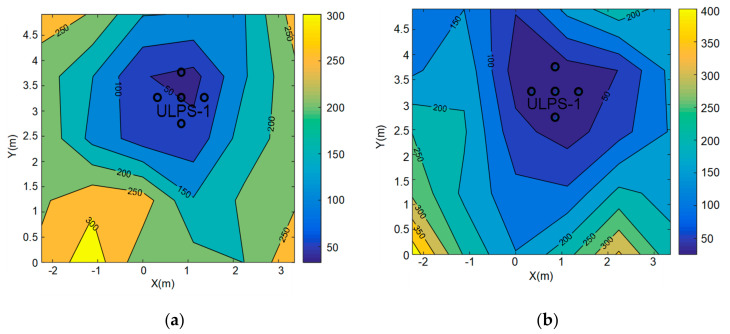
Position dilution of precision (PDOP) representation for ULPS-1: (**a**) at *z*_1_ = 1.35 m; (**b**) at *z*_2_ = 1.93 m.

A final study has been developed with regard to the PDOP for the ULPS-1 at heights *z*_1_ and *z*_2_ in Figure 7. Both heights present a similar range of values, where the lowest PDOP values are below the ULPS-1 and the highest PDOP values are in the proximities of the transducers, as expected. Furthermore, the height *z*_2_ provides lower PDOP values than *z*_1_, as expected as well, since the PDOP increases as the distance between the ULPS and the receiver does.

## 4. Novel Proposal Based on Three ULPSs

After analyzing the behavior of the ULPS-1 operating in an independent way, all the three ULPSs, arranged as described in Section 2, have been simulated together to analyze the behavior in the same points P1–P7 considered for a single ULPS in Section 3. A MLE fusion approach has been applied to merge the position estimates from each ULPS, and a later average for every receiver, as was detailed in Section 2.2 [36].

**Figure 8:**
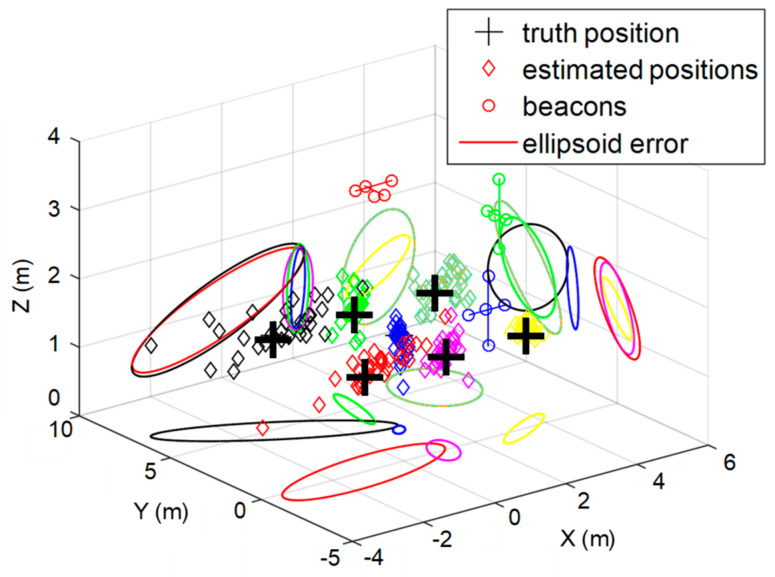
Estimated positions for the considered points (P1–P7) after fusion, including the projections of their corresponding error ellipsoids with a certainty of 95%, at *z*_1_ = 1.35 m.

In the same way as before, two different heights have been simulated (*z*_1_ = 1.35 m and *z*_2_ = 1.93 m), with a set of seven positions in each plane. The estimated positions after fusion present different accuracies, depending on the region in the coverage zone. The position obtained in the intersection of three independent coverage volumes is the best one in terms of accuracy, whereas those areas where only one ULPS is available are the least accurate. Figure 8 depicts the estimated positions after the whole fusion process for *z*_1_ = 1.35 m. Additionally, it is possible to check how only points P1 and P5 (red and black diamonds, respectively) present higher dispersions than the others, mainly due to a longer distance to the ULPSs and/or to a coverage-limited zone.

Furthermore, Table 4 summarizes those results by providing the mean error and the standard deviation per axis for the seven points considered (P1–P7). The lowest mean error and standard deviation, as well as the smallest error ellipsoid, is for P4, the most centered point in the covered volume (blue diamonds in Figure 8). On the other hand, the worst results are obtained for P1 and P5, further away from the central axis of the three ULPSs.

**Figure 9:**
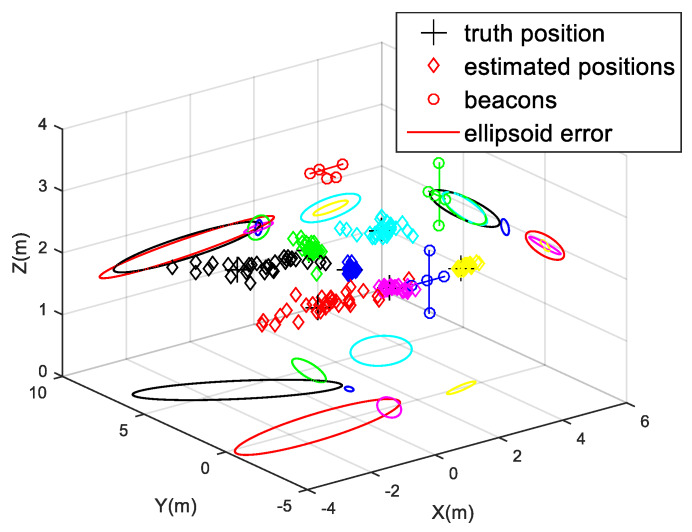
Estimated positions for the considered points (P1–P7) after fusion, including the projections of their corresponding error ellipsoids with a certainty of 95%, at *z*_2_ = 1.93 m.

With regard to the second height considered *z*_2_ = 1.93 m, in general terms, the simulated results obtained after fusion are better compared to those from a single ULPS, as can be observed in Figure 9. Table 5 summarizes these figures again. Similar conclusions can be derived, where P4 (blue diamonds) is still the best one. Compared to the other height *z*_1_ (depicted in Figure 8), the worst performance is still obtained in points P1 and P5 (red and black diamonds, respectively), although it is important to remark that the height *z*_2_ is very close to the one at which the ULPS-2 is installed (see Table 1). This fact provides a significant accuracy in the estimation of the coordinate *z*, with a low dispersion, as it is possible to observe in Figure 9.

**Figure 10:**
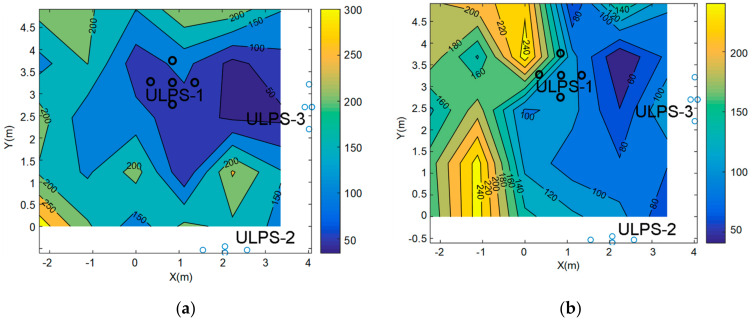
PDOP estimation when merging the three ULPSs at: (**a**) *z*_1_ = 1.35 m and (**b**) *z*_2_ = 1.93 m.

The PDOP estimation has been obtained by simulation for both heights, *z*_1_ and *z*_2_, after fusion and it is depicted in Figure 10. The PDOP values range from 50 to 250 for both heights, where the areas with high values are defined by the lack of one or two ULPSs’ coverage. In both cases, the regions with lower PDOP values correspond to the right areas, where the coverages from the three ULPSs are easily overlapped. Note as well that some areas close to the ULPSs present a reduced coverage, since, sometimes, only one ULPS is available. This constraint may be tackled by including more ULPSs in the assembly or by changing the transducers used as emitters to others with a wider aperture beam. Furthermore, the PDOP value is higher for *z*_2_ at the left corners of the environment, where the coverage from the three ULPSs is more unlikely to be available simultaneously. Note that these zones with limited coverage are bigger as the height increases. Nevertheless, in the central region where all the ULPS are available, the PDOP values at *z*_2_ are still slightly better than at *z*_1_.

In order to compare the accuracy of simulated results before and after MLE fusion, the cumulative distribution function (CDF) has been calculated for all the grid of positions. Table 6 and Table 7 provide the errors for 90% of the cases for the seven considered points (P1–P7) for *z*_1_ and *z*_2_, respectively. It is worth noting that the positioning error of the estimated positions obtained by simulation, before fusion, is in the range of decimeters or even meters, whereas the errors after fusion is in the range of centimeters or decimeters. Apart from the expected differences among the points and the heights, generally speaking, errors improve in all cases if the three ULPSs are merged.

## 5. Experimental Results

**Figure 11:**
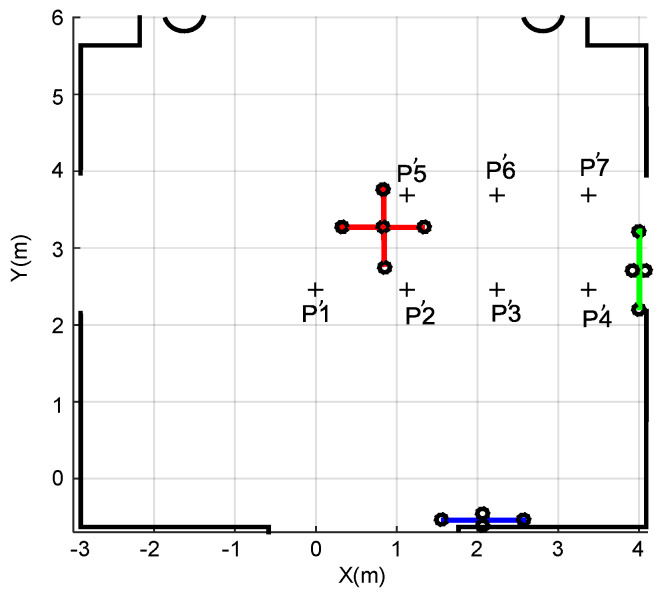
Experimental workspace, including the measurement points (P’1–P’7) at heights *z*_1_ = 1.35 m and *z*_2_ = 1.93 m.

Experimental tests have been carried out for the proposed setup, according to the scenario previously described in Figure 2. The beacons from the three ULPSs are encoded with fifteen different 255-bit Kasami sequences, whereas a multiple ultrasonic receiver prototype has been used. Figure 11 depicts a scheme about the workspace, where the three ULPSs have been installed to cover an approximated volume of 196 m^3^. A second set of seven measurement points (P’1–P’7) has been considered here, at the same heights *z*_1_ = 1.35 m and *z*_2_ = 1.93 m. Thirty measurements have been obtained at each point. The reason why another set of points has been selected is to match better the actual coverage of all the ULPS. Nevertheless, the simulations corresponding to these new points are also included hereinafter for comparison between real and simulated results. We also include the real results obtained in these points in the case of using only one ULPS (e.g., ULPS-1) to highlight the differences regarding the use of three ULPSs. The experimental setup calibration, as well as the ground-truth determination, have been carried out manually, by means of a laser plumb (for angles) and a laser distance meter (for distances).

**Figure 12:**
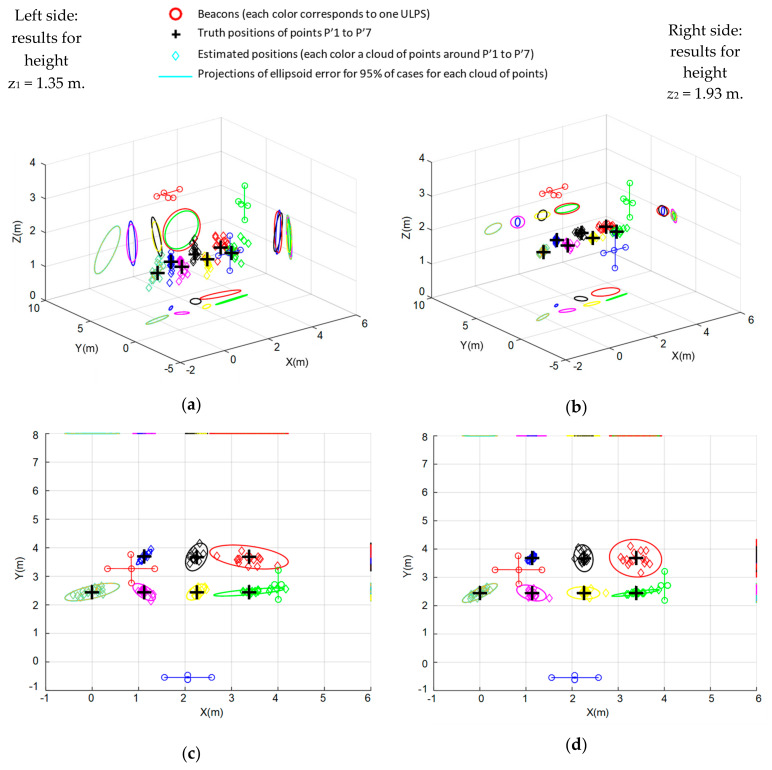

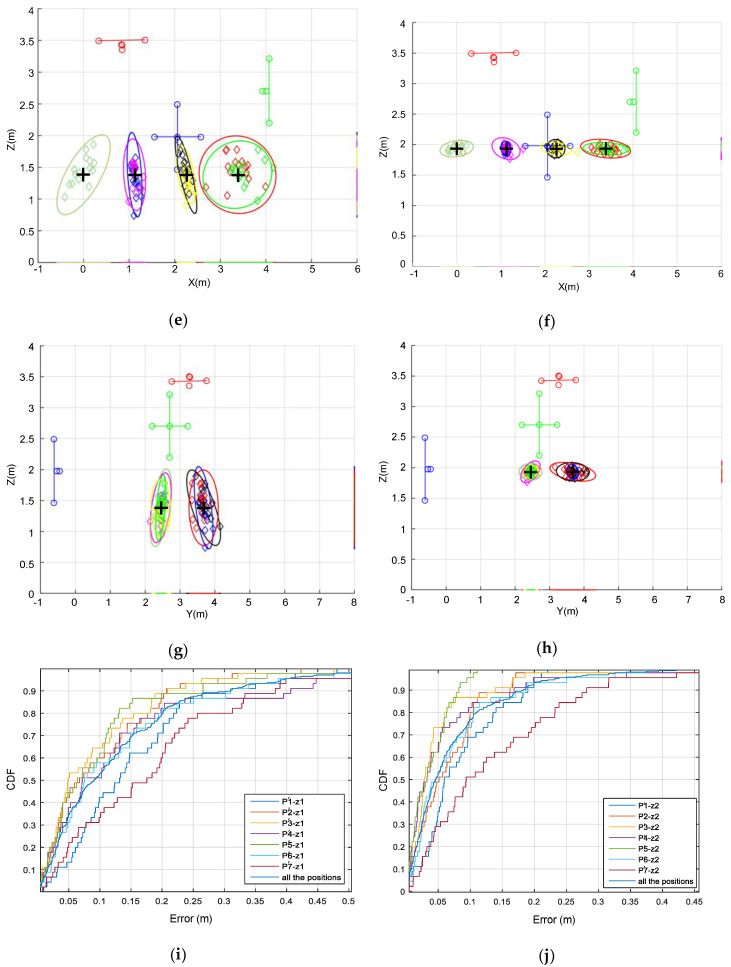
Experimental results for the test points (P’1–P’7) for both heights, *z*_1_ = 1.35 m on the left and *z*_1_ = 1.93 m on the right: (**a**,**b**) 3D representation of clouds of points; (**c**,**d**) Y-X projections; (**e**,**f**) Z-X projections; (**g**,**h**) Z-Y projections; (**i**,**j**) experimental CDFs for the results at each test point and for all of them. All the cases include the projections of their corresponding error ellipsoids with a certainty of 95%, after the average and the maximum likelihood estimation (MLE) fusion.

It is worth mentioning that all the ULPSs are detected at all the test points (P’1–P’7). Figure 12 plots all these experimental results after applying the fusion algorithm. It has been arranged to easily compare results at both heights: *z*_1_ = 1.35 m corresponds to all the subplots on the left and *z*_2_ = 1.93 m to those on the right. Successive rows include the following: Figure 12a,b a 3D representation of the clouds of points obtained around every test point (P’1–P’7), as well as the projections of their corresponding uncertainty ellipsoids on the three coordinate planes; Figure 12c–h are the projections of the clouds of points on the coordinate planes Y-X, Z-X and Z-Y; Figure 12i,j include the CDFs for the positioning error corresponding to the results obtained at each test point and for all of them. Note that results for *z*_2_ = 1.93 m are better, as the test points are nearer to the ULPSs and more centered with respect to their coverage area. In fact, the dispersion of results is greater as the test points are further from the center of the coverage area. Regarding the CDFs, it is straighforward to conclude that the performance of points P’3, P’4 and P’5 are the best, followed by P’6 and P’2 and, finally, P’1 and P’7 (the last is the worst due to its bad coverage from all the ULPSs). Furthermore, note that, on average for all the test points, errors are always below 0.26 m for *z*_1_ = 1.35 m and below 0.19 m for *z*_2_ = 1.93 m, in both cases for 90% of the cases.

For the sake of numerical comparison, Table 8 and Table 9 show the positioning errors for each coordinate, for all the test points at *z*_1_ = 1.35 m and for real and simulated measurements, respectively. Although there are real effects that are not considered in simulations (as multipath and noises), there is an agreement in the distribution of the errors according to the position of the test point. Similar conclusions can be derived from data for test points at *z*_2_ = 1.93 m, shown in Table 10 and Table 11, for real and simulated results, respectively.

**Figure 13:**
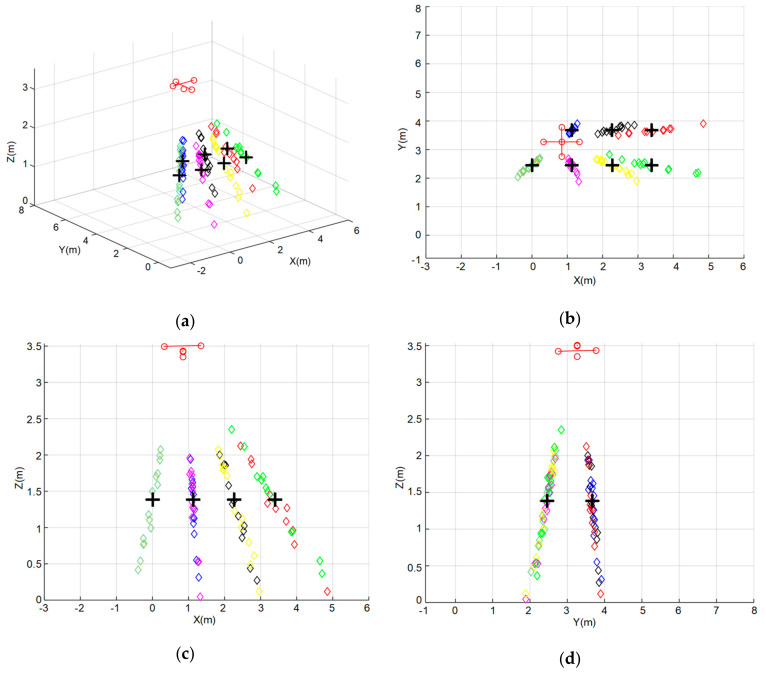

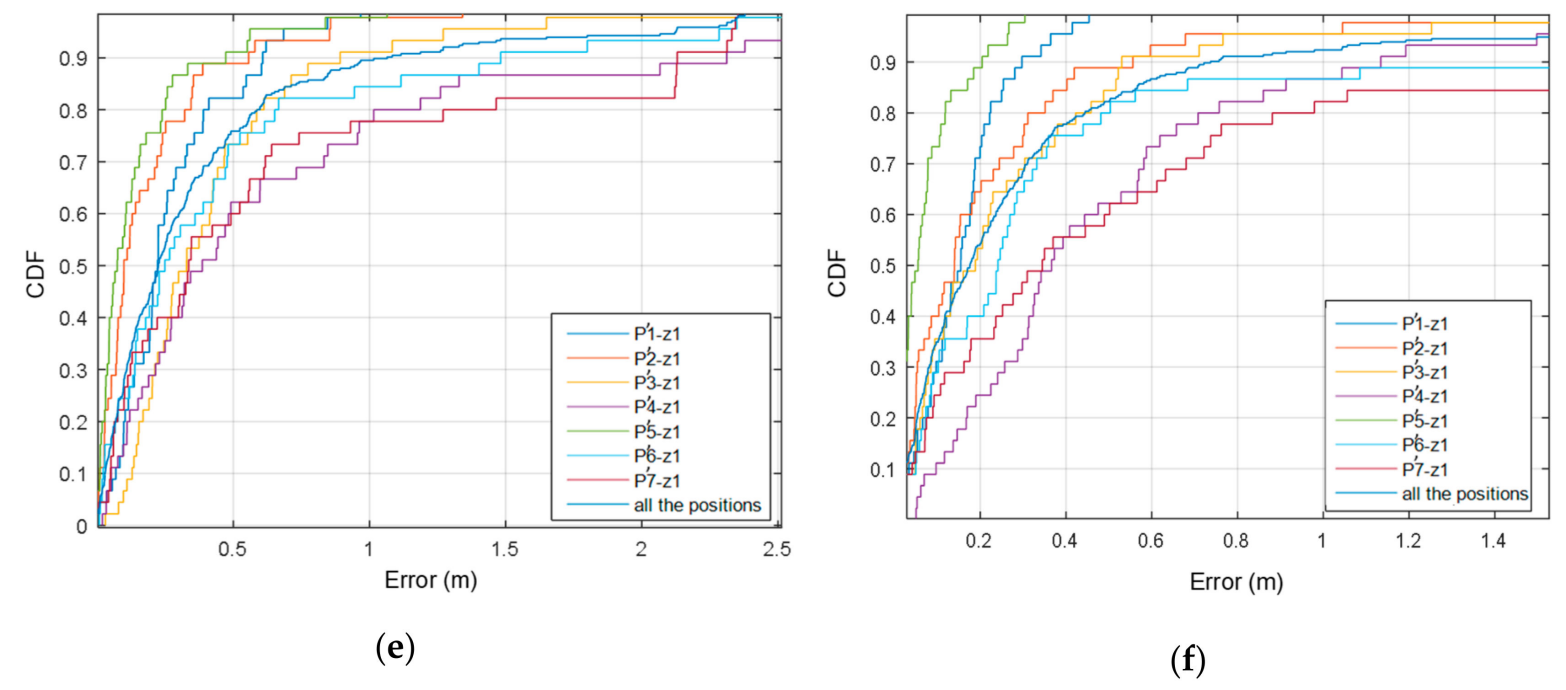
Results obtained in the same test points (P’1–P’7) in the case of using only ULPS-1: (**a**–**e**) are the cloud of points and the error CDF for the test-points at *z*_1_ = 1.35 m, whereas (**f**) shows the error CDF at *z*_1_ = 1.93 m.

To highlight the differences between obtaining the position with only one ULPS and with the fusion of all of them, Figure 13 shows the results on the same test points (P’1–P’7) in the case that the positions are estimated using only measurements from ULPS-1: Figure 13a–d corresponds to *z*_1_ = 1.35 m; the corresponding error CDF is presented in Figure 13e; and, additionally, Figure 13f shows the error CDF for the test points at *z*_2_ = 1.93 m.

Comparing Figure 12 and Figure 13 for both heights, *z*_1_ and *z*_2_, the improvement is clear. For instance, in the error CDFs, considering all the test-points, for 90% of the cases errors are below 1.1 m at *z*_1_ = 1.35 m and below 0.75 m for *z*_2_ = 1.93 m (in the case of fusion these values were below 0.26 m for *z*_1_ = 1.35 m and below 0.19 m for *z*_2_ = 1.93 m). 

Table 12 (at *z*_1_ = 1.35 m) and Table 13 (at *z*_1_ = 1.93 m) extend these comparisons of errors for 90% of the cases, between the results obtained using only one ULPS and using the fusion from all of them. 

In summary, the performance of the proposal for both considered heights, *z*_1_ and *z*_2_, is similar, resulting in a better estimation for coordinate *z* at *z*_2_. It can be observed how the fusion improves performance at both heights; the positioning error, for 90% of the cases, remains below 20 cm roughly for all the central test points (at planes *z*_1_ and *z*_2_) and below 28 cm for all cases in plane *z*_2_ and around 30 cm in plane *z*_1_ (with the exception of point P’7 that increases to 38 cm). It is important to point out that these are maximum errors for 90% of the cases and not average errors, which, as can be observed in Table 8, Table 9, Table 10 and Table 11, are below 10 cm in most cases.

Concerning further comparisons with other previous works, it is difficult to find exactly the same or similar experimental setups in the literature. In some cases, the dimensions and complexity of the area under exploration are lower, the type of transducers can be different and might influence the final performance, the ultrasonic transmissions can be narrow band, instead of the wide band presented here, the modulation scheme or the sequences applied in the encoding techniques have a definitive importance on the final achieved errors, or even the inclusion of a synchronism link between emitters and receivers. Taking into account these considerations, we have compared the solution with only one LPS (Section 3) and the proposal with three ULPSs (Section 4), particularly in Figure 12 and Figure 13. Furthermore, in [30] the single LPS has been experimentally characterized in the same environment with errors around 20 cm. With regard to some recent previous works, in [37] a system based on pseudo-random signals and a smartphone as receiver is proposed for 3D positioning, achieving errors in the range of some decimeters. Another solution is proposed in [38], dealing with Kasami sequences and with errors in the range of decimeters for a smaller coverage area. In general terms, it is possible to observe a decrease in the positioning error whether more beacons are installed in the environment and the corresponding measurements are merged as has been proposed in this work.

## 6. Conclusions

In this work, a 3D positioning system based on ultrasounds has been analyzed and experimentally tested in a real scenario. It is based on the deployment of three ultrasonic beacon units (ULPS) that operate independently, as well as on an ultrasonic receiving prototype composed by three transducers. This prototype can determine the time-differences-of-arrival (TDOA) for every transmitter from each beacon unit and then obtain up to three different position estimates, each one per ULPS available. These three independent position estimates are merged together by using a MLE fusion algorithm. Since the three ULPSs are installed on orthogonal planes, they are complementary in order to compensate for the low accuracy observed in the coverage zone for the perpendicular direction to the each ULPS. This allows the final positioning errors to be decreased. The 3D arrangement and proposal have been successfully validated in a large university hall, confirming that the proposal can be applied to positioning targets with high accuracy in 3D volumes with an adequate coverage. The obtained accuracies are in a range of decimeters for 90% of the cases in the central zone of the room, where the transmissions from the three beacon units are available at the same time (this could be suitable for applications such as guiding mobile robots or tracking some parts of a person’s body). On the other hand, these accuracies can increase above half a meter when, either the point under analysis is far away from the beacon units, or the coverage from one or more beacon units is missing (this accuracy could be enough for people guidance anyway). In order to achieve a uniform distribution of the positioning error in all the experimental scenarios, other ULPSs should be deployed.

## Figures and Tables

**Table 1:** Coordinates of the central beacon B1 for every ULPS in the considered workspace in Figure 1.

ULPS	Coordinates for B1 (m)
ULPS1	(0.84, 3.267, 3.351)
ULPS2	(2.06, −0.458, 1.980)
ULPS3	(3.92, 2.7, 2.7)

**Table 2:** Mean positioning errors and standard deviations for points P1–P7 at *z*_1_ = 1.35 m.

Mean Error (m)				Standard Deviation (m)		
Points	x	y	z	x	y	z
P1	1.091	1.819	1.187	0.908	1.553	0.970
P2	0.083	0.787	0.533	0.066	0.964	0.596
P3	1.416	1.798	1.242	1.484	1.949	1.314
P4	0.053	0.158	0.380	0.056	0.190	0.487
P5	0.745	0.628	0.891	0.532	0.451	0.667
P6	0.086	0.450	0.599	0.088	0.458	0.560
P7	4.539	4.946	1.287	2.040	1.308	1.132

**Table 3:** Mean positioning errors and standard deviations for points P1–P7 at *z*_1_ = 1.93 m.

Mean Error (m)				Standard Deviation (m)		
Points	x	y	z	x	y	z
P1	0.713	1.207	0.753	0.611	1.004	0.607
P2	0.118	1.170	0.575	0.119	1.166	0.562
P3	1.219	1.579	0.996	0.690	0.877	0.589
P4	0.040	0.132	0.259	0.033	0.121	0.231
P5	0.648	0.544	0.563	0.796	0.645	0.670
P6	0.061	0.410	0.412	0.054	0.454	0.436
P7	4.607	4.988	0.535	1.264	0.840	0.563

**Table 4:** Mean positioning errors and standard deviations for points P1–P7 at *z*_1_ = 1.35 m.

Mean Error (m)				Standard Deviation (m)		
Points	x	y	z	x	y	z
P1	0.503	0.046	0.220	0.780	0.520	0.281
P2	0.035	0.140	0.107	0.152	0.419	0.331
P3	0.025	0.023	0.022	0.227	0.166	0.100
P4	0.005	0.006	0.051	0.093	0.107	0.105
P5	0.066	0.002	0.080	0.761	0.664	0.246
P6	0.014	0.012	0.035	0.086	0.460	0.183
P7	0.031	0.282	0.088	0.400	0.94	0.260

**Table 5:** Mean positioning errors and standard deviations for points P1–P7 at *z*_2_ = 1.93 m.

Mean Error (m)				Standard Deviation (m)		
Points	x	y	z	x	Y	z
P1	0.482	0.001	0.016	0.716	0.432	0.050
P2	0.056	0.031	0.008	0.121	0.159	0.017
P3	0.013	0.024	0.001	0.158	0.058	0.021
P4	0.001	0.034	0.010	0.034	0.105	0.041
P5	0.423	0.204	0.002	1.324	1.047	0.077
P6	0.012	0.029	0.017	0.082	0.288	0.049
P7	0.082	0.288	0.049	0.398	0.583	0.093

**Table 6:** Cumulative distribution function (CDF) positioning error for 90% of estimated positions at *z*_1_ = 1.35 m.

Positions	Before Fusion (m)			After Fusion (m)
	ULPS-1	ULPS-2	ULPS-3	3 ULPSs
P1	2.499	1.875	3.288	1.071
P2	2.357	0.941	2.338	0.540
P3	3.497	0.71	1.191	0.504
P4	0.438	0.75	1.639	0.174
P5	1.543	4.43	2.035	1.256
P6	0.716	1.431	1.639	0.432
P7	7.047	4.684	5.26	1.163

**Table 7:** CDF positioning error for 90% of estimated positions at *z*_2_ = 1.93 m.

Positions	Before Fusion (m)			After Fusion (m)
	ULPS-1	ULPS-2	ULPS-3	3 ULPSs
P1	2.314	1.089	4.547	0.99
P2	1.828	0.501	2.448	0.235
P3	2.531	0.216	1.4	0.205
P4	0.455	1.123	0.967	0.119
P5	1.688	3.189	2.96	1.395
P6	0.465	1.693	2.769	0.455
P7	5.134	5.286	5.181	0.804

**Table 8:** Real results at *z*_1_ = 1.35 m. Mean and standard deviation of errors in each coordinate for all the test points.

Positions	Mean Error (m)			Std Deviation (m)		
	X(m)	Y(m)	Z(m)	X(m)	Y(m)	Z(m)
P’1	0.047	0.046	0.055	0.072	0.070	0.125
P’2	0.014	0.087	0.059	0.017	0.090	0.047
P’3	0.042	0.458	0.052	0.027	0.248	0.066
P’4	0.194	0.346	0.020	0.143	0.329	0.066
P’5	0.025	0.062	0.226	0.077	0.317	0.179
P’6	0.064	0.631	0.005	0.051	0.738	0.068
P’7	0.401	0.756	0.138	0.233	0.901	0.140

**Table 9:** Simulated results at *z*_1_ = 1.35 m. Mean and standard deviation of errors in each coordinate for all the test points.

Positions	Mean Error (m)			Std Deviation (m)		
	X(m)	Y(m)	Z(m)	X(m)	Y(m)	Z(m)
P’1	0.0258	0.0144	0.0613	0.0974	0.0571	0.1381
P’2	0.0087	0.0141	0.0154	0.0586	0.0787	0.1025
P’3	0.0390	0.0085	0.1110	0.0587	0.0590	0.1358
P’4	0.1889	0.0289	0.0382	0.2341	0.0372	0.1226
P’5	0.0140	0.0194	0.0815	0.0404	0.0673	0.1582
P’6	0.0274	0.0389	0.1133	0.0527	0.1240	0.1499
P’7	0.0148	0.0918	0.0953	0.1899	0.1180	0.1234

**Table 10:** Real results at *z*_2_ = 1.93 m. Mean and standard deviation of errors in each coordinate for all the test points.

Positions	Mean Error (m)			Std Deviation (m)		
	X(m)	Y(m)	Z(m)	X(m)	Y(m)	Z(m)
P’1	0.643	0.112	0.250	0.347	0.072	0.101
P’2	0.762	0.112	0.250	0.347	0.072	0.101
P’3	0.022	0.167	0.019	0.019	0.306	0.031
P’4	0.167	0.296	0.117	0.255	0.474	0.047
P’5	0.161	0.601	0.032	0.088	0.240	0.023
P’6	0.048	0.323	0.045	0.034	0.403	0.021
P’7	0.383	1.129	0.052	0.296	0.942	0.012

**Table 11:** Simulated results at *z*_2_ = 1.93 m. Mean and standard deviation of errors in each coordinate for all the test points.

Positions	Mean Error (m)			Std Deviation (m)		
	X(m)	Y(m)	Z(m)	X(m)	Y(m)	Z(m)
P’1	0.0040	0.0153	0.0014	0.0620	0.0648	0.0254
P’2	0.0340	0.0405	0.0009	0.0931	0.0566	0.0436
P’3	0.0715	0.0176	0.0023	0.1201	0.0595	0.0221
P’4	0.0710	0.0095	0.0085	0.1368	0.0220	0.0329
P’5	0.0047	0.0075	0.0163	0.0229	0.0375	0.0289
P’6	0.0122	0.0382	0.0010	0.0482	0.1134	0.0338
P’7	0.0081	0.0263	0.0156	0.0869	0.1439	0.0306

**Table 12:** Positioning error containing 90% of the estimated positions at *z*_1_ = 1.35 m.

Points	Before Fusion (m)			After Fusion (m)
	ULPS-1	ULPS-2	ULPS-3	3 ULPSs
**P’1**	0.608	2.061	1.383	0.310
**P’2**	0.551	1.165	1.246	0.208
**P’3**	0.892	0.872	1.010	0.208
**P’4**	2.313	0.998	0.823	0.206
**P’5**	0.472	0.788	2.578	0.265
**P’6**	1.482	1.165	1.010	0.310
**P’7**	2.130	1.863	0.679	0.381

**Table 13:** Positioning error containing 90% of the estimated positions at *z*_2_ = 1.93 m.

Points	Before Fusion (m)			After Fusion (m)
	ULPS-1	ULPS-2	ULPS-3	3 ULPSs
**P’1**	0.297	3.318	4.367	0.189
**P’2**	0.556	1.012	1.293	0.162
**P’3**	0.53	0.762	0.494	0.139
**P’4**	1.134	1.057	0.187	0.168
**P’5**	0.204	2.072	1.091	0.082
**P’6**	1.686	1.65	0.527	0.186
**P’7**	2.049	1.19	0.542	0.281
